# Investigating the Process of Autoimmune Inner Ear Disease: Unveiling the Intricacies of Pathogenesis and Therapeutic Strategies

**DOI:** 10.7150/ijms.97831

**Published:** 2025-01-01

**Authors:** Mengmeng Wang, Ping Zhang, Qiang Li, Chunyu Kong

**Affiliations:** 1 Department of Rheumatism and Immunology, Tianjin First Central hospital, Tianjin, China.; 2 Department of Pharmacy, Tianjin Union Medical Center, Tianjin, China.

**Keywords:** Autoimmune Inner Ear Disease, Sensorineural Hearing Loss, Immunopathology, Autoantibodies, Immunosuppressive Therapy

## Abstract

Autoimmune inner ear disease (AIED) is a rare condition characterized by immune-mediated damage to the inner ear, leading to progressive sensorineural hearing loss (SNHL) and vestibular symptoms such as vertigo and tinnitus. This study investigates the pathogenesis and therapeutic strategies for AIED through the analysis of three cases with different underlying autoimmune disorders: rheumatoid arthritis, relapsing polychondritis, and IgG4-related disease. The etiology of AIED involves complex immunopathological mechanisms, including molecular mimicry and the "bystander effect," with specific autoantibodies, such as those against heat shock protein 70 (HSP70), playing a potential role in cochlear damage. Diagnosis remains challenging due to nonspecific symptoms and the lack of distinct biomarkers, emphasizing the need for comprehensive clinical evaluation and exclusion of other hearing loss causes. Treatment primarily involves immunosuppressive therapies, with glucocorticoids as the first line, effective in 70% of cases. However, resistance or partial response necessitates the use of additional agents like methotrexate and biologics such as anti-TNF and IL-6 receptor antagonists. Early intervention is crucial for favorable outcomes, as demonstrated in the studied cases, where timely corticosteroid and immunosuppressive treatments led to significant hearing improvement. The study underscores the importance of personalized treatment strategies based on individual immunologic profiles and comorbidities. Our findings highlight the heterogeneity of AIED and the potential for biologic therapies in refractory cases.

## Introduction

Autoimmune inner ear disease (AIED) is a rare but clinically significant condition characterized by immune-mediated damage to the inner ear, leading to progressive sensorineural hearing loss (SNHL) and often accompanied by vestibular symptoms such as vertigo and tinnitus 1, 2. AIED can present as an isolated autoimmune disorder targeting inner ear structures or as a secondary manifestation associated with systemic autoimmune diseases like rheumatoid arthritis, systemic lupus erythematosus, and Cogan's syndrome 3, 4. The clinical progression of AIED varies among patients, with some exhibiting rapid deterioration in hearing and others showing fluctuation in symptoms, often confounding timely diagnosis and treatment 5.

The etiology of AIED remains incompletely understood, though several immunopathological mechanisms have been proposed. A key theory posits the role of the inner ear's immune system, particularly within the endolymphatic sac, in generating and sustaining an immune response against self-antigens following an initial injury or inflammatory insult 6. This response may be perpetuated through molecular mimicry, where antibodies developed against pathogens with antigenic similarity to inner ear proteins cross-react with host tissues 7. Other hypotheses include the “bystander effect,” in which cytokines such as interleukin-1 (IL-1) and tumor necrosis factor (TNF) released from adjacent cells promote local immune activation, and immune tolerance breakdown, wherein concealed antigens become accessible to the immune system upon tissue damage, provoking an autoimmune response 8.

Various autoantibodies, both organ-specific and non-specific, have been implicated in the pathogenesis of AIED 5, 9. Specific antibodies that target inner ear antigens include those against collagen type II and IX, cochlin, and heat shock protein 70 (HSP70), which may play a pivotal role in recognizing and damaging cochlear and vestibular cells 10. Notably, antibodies against HSP70 have been identified in AIED patients with high sensitivity (54.5%) and specificity (42.9%), suggesting HSP70 as a potential biomarker for early AIED detection. The cross-reactivity of these autoantibodies with homologous proteins on inner ear cells has been proposed as a mechanism driving chronic inflammation and tissue destruction, resulting in sensorineural damage. In addition to specific autoantibodies, non-specific antibodies, including antinuclear antibodies (ANA), rheumatoid factor (RF), and antiphospholipid antibodies, are frequently detected, particularly in cases where AIED coexists with systemic autoimmune diseases 11.

Clinically, AIED is challenging to diagnose due to its nonspecific presentation and the absence of pathognomonic signs. Diagnostic criteria, as established at the 1994 National AIED Conference, emphasize the necessity of a history of rapidly progressive, often fluctuating bilateral or unilateral SNHL, potentially accompanied by vestibular symptoms such as dizziness or tinnitus, and the presence of autoimmune serological markers 12. Differential diagnosis is essential, requiring exclusion of other common etiologies of hearing loss, including sudden sensorineural hearing loss, ototoxicity, and presbycusis.

Treatment strategies for AIED have largely centered around immunosuppressive therapies. Glucocorticoids are considered first-line treatment due to their effectiveness in reducing inflammation and stabilizing hearing in approximately 70% of AIED cases 5. However, corticosteroid resistance or partial response can occur, especially with delayed initiation of therapy. Furthermore, the need for long-term corticosteroids carries the risk of significant adverse effects, making steroid-sparing agents like methotrexate, azathioprine, and mycophenolate mofetil valuable adjuncts in prolonged disease management. Recently, biologics such as anti-TNF agents, IL-6 receptor antagonists, and rituximab have shown promise in refractory cases, though more extensive studies are required to validate their efficacy and safety in AIED.

Given the autoimmune basis and variable response to treatment in AIED, a nuanced understanding of each patient's immunologic profile and comorbid conditions is crucial. Studies have indicated that AIED patients with shorter disease duration and less severe hearing impairment at presentation are more likely to achieve favorable auditory outcomes with timely immunosuppressive therapy. We recently observed three sample of AIED with differing underlying autoimmune disorders: rheumatoid arthritis, recurrent polychondritis, and IgG4-related disease. Each case demonstrated sensorineural hearing loss primarily affecting high frequencies, with varying degrees of improvement following immunosuppressive treatment, thus underscoring the heterogeneity.

## Methods

### Ethical Statement

All procedures performed in studies involving human participants were in accordance with the ethical standards of the institutional and national research committee and with the 1964 Helsinki declaration and its later amendments or comparable ethical standards. Approval was granted by the Ethics Review Board of the Tianjin First Central hospital (No. 221RS).

### Pure Tone Audiometry

Audiometric testing was conducted using an LK225 pure-tone audiometer to assess participants' hearing levels. Calibration of the audiometer was performed as per manufacturer instructions, and each participant's ear canals were examined to ensure no obstructions, such as cerumen, were present 13. Participants were instructed to press the response button upon hearing any sound. For air conduction, testing began at 500 Hz with a stimulus intensity of 60 dB, adjusting intensity incrementally to determine each participant's threshold. The process was repeated at frequencies of 125 Hz, 250 Hz, 1000 Hz, 2000 Hz, 4000 Hz, and 8000 Hz. Bone conduction testing was conducted by placing a transducer on the mastoid bone of the test ear, using the same threshold-determination method. If an air conduction threshold difference exceeding 40 dB was observed between ears, or if there were discrepancies in bone conduction thresholds, masking noise was applied to the non-test ear to improve measurement accuracy 14.

### Anti-Nuclear Antibody (ANA) Detection

For the detection of anti-nuclear antibodies (ANA), a Zeus Scientific ANA Detection Kit was used in conjunction with the Luminex 200 Athena flow cytometry system, employing a multiplex microbead flow immunofluorescence assay. Reagents were equilibrated to room temperature (20-25°C) out of direct light. Negative control was assigned to well A1, with positive controls in wells B1-E1 15. Microbead suspension was vortexed for 30 seconds and ultrasonicated for another 30 seconds to ensure homogeneity. In a separate dilution plate, serum samples and controls were diluted 1:21 (10 µL serum to 200 µL diluent). Fifty microliters of the microbead suspension and 10 µL of each diluted sample or control were added to designated wells. Following a 30 ± 10-minute room temperature incubation, the plate was washed using a vacuum pump and 200 µL wash solution, repeated thrice, and left to air dry for 3-5 minutes. Afterward, 150 µL of binding reagent was added, followed by another 30-minute incubation before analysis on the Luminex 200 Athena system, with results recorded within 60 minutes of incubation 16.

### Rheumatoid Factor (RF) Detection

Rheumatoid factor (RF) levels were measured using an AESKU RF Detection Kit with the TECAN SUNRISE ELISA reader. The concentrated diluent was first diluted five-fold with distilled water, then the serum samples were further diluted 1:101. The plate was washed thrice with 300 µL wash buffer per well 17. Diluted patient samples (100 µL) and controls were added to designated wells and incubated at 20-32°C for 30 minutes. After washing, 100 µL enzyme conjugate was added, incubated for another 30 minutes, and washed. Finally, 100 µL of TMB substrate was added for a 30-minute dark incubation, followed by 100 µL stop solution. Absorbance was read at 450 nm using the TECAN SUNRISE reader 18.

### Anti-Cyclic Citrullinated Peptide Antibody (ACCP) Detection

The detection of anti-cyclic citrullinated peptide antibodies (ACCP) was carried out using a Roche ACCP Antibody Detection Kit with an electrochemiluminescence analyzer. All kit reagents were loaded into the analyzer, and the device automatically calculated the analyte concentration for each sample 19.

### Anti-Neutrophil Cytoplasmic Antibody (ANCA) Detection

Anti-neutrophil cytoplasmic antibodies (ANCA) against proteinase 3 and myeloperoxidase were measured using the Zeus Scientific ANCA Detection Kit and the Luminex 200 Athena system, following a protocol identical to that of the ANA assay 20.

### IgG4 Detection

IgG4 levels were determined using a Siemens IgG4 Detection Kit through a turbidimetric immunoassay on the BN II automated protein analyzer. Serum samples were diluted 1:2000, and the BN system handled all subsequent steps 21.

### Statistical Analysis

Statistical analyses were performed using SPSS (version 25.0). Descriptive statistics were calculated for all quantitative variables, presented as mean ± standard deviation. Group comparisons were analyzed using independent t-tests for normally distributed data and Mann-Whitney U tests for non-normal distributions 22. Chi-square or Fisher's exact tests were used for categorical data 23. A p-value of less than 0.05 was considered statistically significant. Sensitivity, specificity, and other diagnostic metrics were computed where relevant, with Pearson or Spearman correlation coefficients employed for correlation analyses based on data normality.

## Results

### Rheumatoid Arthritis with Bilateral High-Frequency Sensorineural Hearing Loss

A 43-year-old female was admitted with complaints of bilateral hearing loss progressing over two months and a 15-year history of rheumatoid arthritis primarily affecting the temporomandibular joints, proximal interphalangeal, and metacarpophalangeal joints. The patient reported high-pitched tinnitus and episodic dizziness, which intensified with positional changes, but denied any otalgia or otorrhea. Otoscopic examination revealed clear external auditory canals and intact tympanic membranes without tenderness over the mastoid area. Physical examination showed ulnar deviation of the hands with “swan neck” deformities and tenderness in multiple joints.

**Figure 1:**
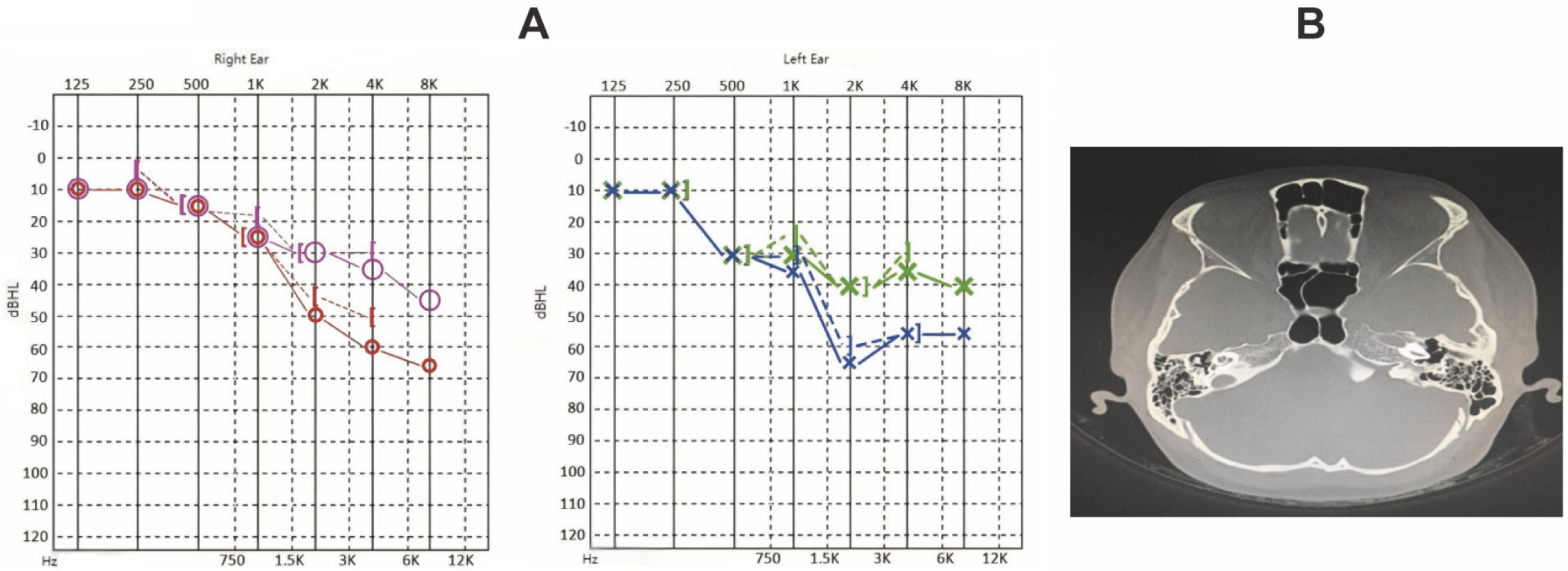
** The characterization of case-1.** A. Hearing curves before and after treatment for Case 1 (red and blue represent pre-treatment, pink and green represent post-treatment). B. Temporal bone CT findings for Case 1 show bilateral well-aerated mastoid air cells, clear bilateral tympanic cavity and mastoid antrum, and absence of apparent abnormal density lesions. Intact ossicular chain with no significant abnormalities in morphology and bone structure of auditory ossicles. No significant abnormalities observed in bony structures of bilateral inner ears.

Laboratory tests indicated elevated rheumatoid factor (>300 U/mL; normal <18 U/mL), antinuclear antibody (ANA) titer of 1:320, anti-cyclic citrullinated peptide (ACCP) antibody (286.1 U/mL; normal <17 U/mL), high-sensitivity C-reactive protein (hs-CRP) at 88.15 mg/L (normal 0-5 mg/L), and erythrocyte sedimentation rate (ESR) of 103 mm/h (normal 0-20 mm/h). Pure tone audiometry demonstrated bilateral sensorineural hearing loss, predominantly affecting high frequencies. Right ear air conduction thresholds (PTA for 500, 1000, 2000 Hz) were 30 dB, and bone conduction was 28 dB. The left ear showed air conduction thresholds of 43 dB and bone conduction of 40 dB. Temporal bone CT imaging showed no structural abnormalities (Figure 1A-B) 24.

Following the American College of Rheumatology/European League Against Rheumatism (ACR/EULAR) 2009 criteria, a diagnosis of rheumatoid arthritis was confirmed, with a Disease Activity Score 28 (DAS28) of 7.97, indicating high disease activity. The patient was started on prednisone 15 mg daily, which was tapered over 12 weeks, along with leflunomide and loxoprofen for disease modification and symptom control. By the 12-week follow-up, hs-CRP and ESR levels had decreased, and the patient reported improvement in tinnitus frequency and dizziness. Audiometry results indicated a significant improvement, particularly in high-frequency thresholds, with right ear air conduction thresholds at 23 dB and left ear thresholds at 33 dB.

### Relapsing Polychondritis with Asymmetric Sensorineural Hearing Loss

A 58-year-old male presented with recurrent episodes of right auricular erythema, swelling, and pain over the previous year, initially diagnosed as auricular infection and treated with antibiotics with partial relief. Four months before admission, the patient developed progressive bilateral hearing loss. Physical examination revealed erythematous and swollen right auricle with partial cartilage collapse, patent ear canals, and intact tympanic membranes.

**Figure 2:**
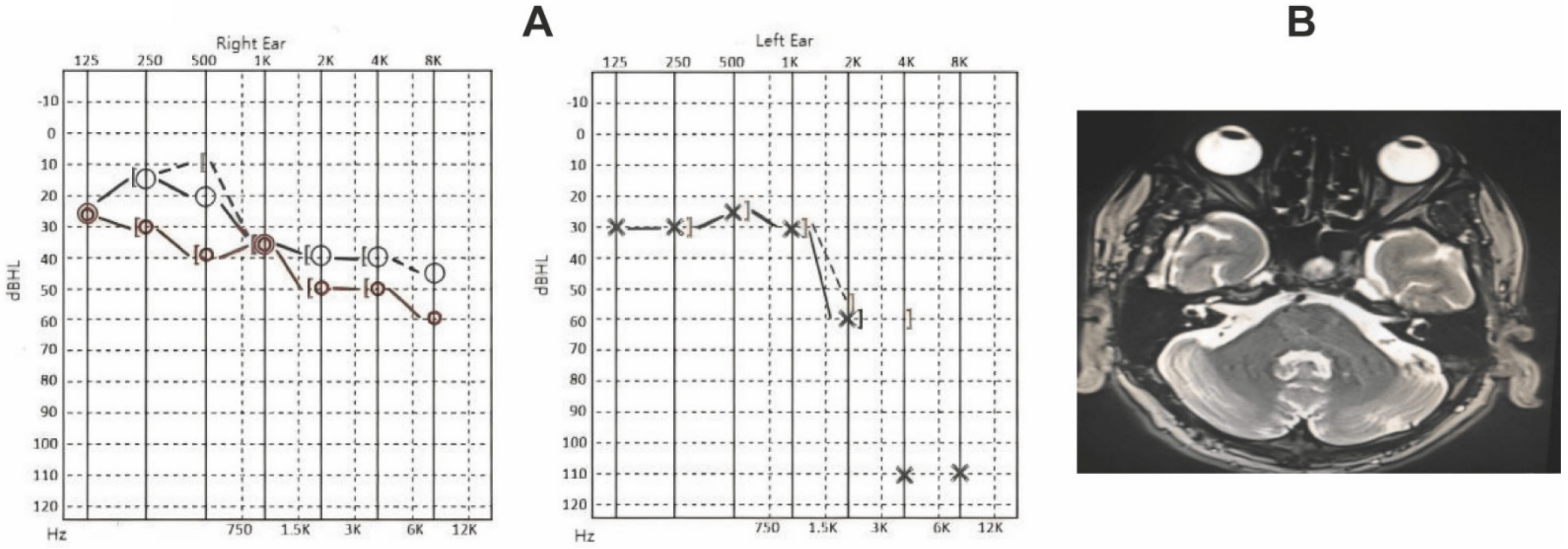
** The characterization of case-2.** A. Hearing curves before and after treatment for Case 2 (red and blue represent pre-treatment, pink and green represent post-treatment). B. Skull base MRI findings for Case 2 show bilateral patent external auditory canals, normal structures of middle ear tympanic cavity, mastoid sinuses, and mastoid antrum, intact bilateral ossicular chains, intact facial nerve canal structure, and normal structures of bilateral semicircular canals and cochlea in the inner ears.

Laboratory testing showed negative ANA and anti-neutrophil cytoplasmic antibody (ANCA) results, hs-CRP of 7.10 mg/L, and ESR of 28 mm/h. Audiometric evaluation demonstrated bilateral sensorineural hearing loss with an asymmetric pattern; right ear air and bone conduction thresholds were 42 dB, while the left ear showed a “sloping” audiometric pattern with profound high-frequency loss. Temporal bone MRI showed patent external auditory canals and normal middle ear structures (Figure 2A-B) 25.

Given the relapsing polychondritis diagnosis and inner ear involvement, the patient was treated with intravenous methylprednisolone (40 mg daily) and azathioprine (100 mg daily). After a week, the steroid dose was gradually tapered. Follow-up audiometry after 14 weeks showed improved right ear hearing thresholds (air conduction PTA: 32 dB), although the left ear demonstrated no significant improvement in high-frequency hearing.

### IgG4-Related Disease with Symmetric Sensorineural Hearing Loss

A 43-year-old female was admitted due to bilateral submandibular gland enlargement persisting for six months, accompanied by hearing loss over the preceding month. Physical examination revealed firm, enlarged submandibular glands (left: 3×4 cm; right: 2×3 cm) with no tenderness or fixation to adjacent tissues. Otoscopic examination indicated clear ear canals and intact tympanic membranes.

**Figure 3:**
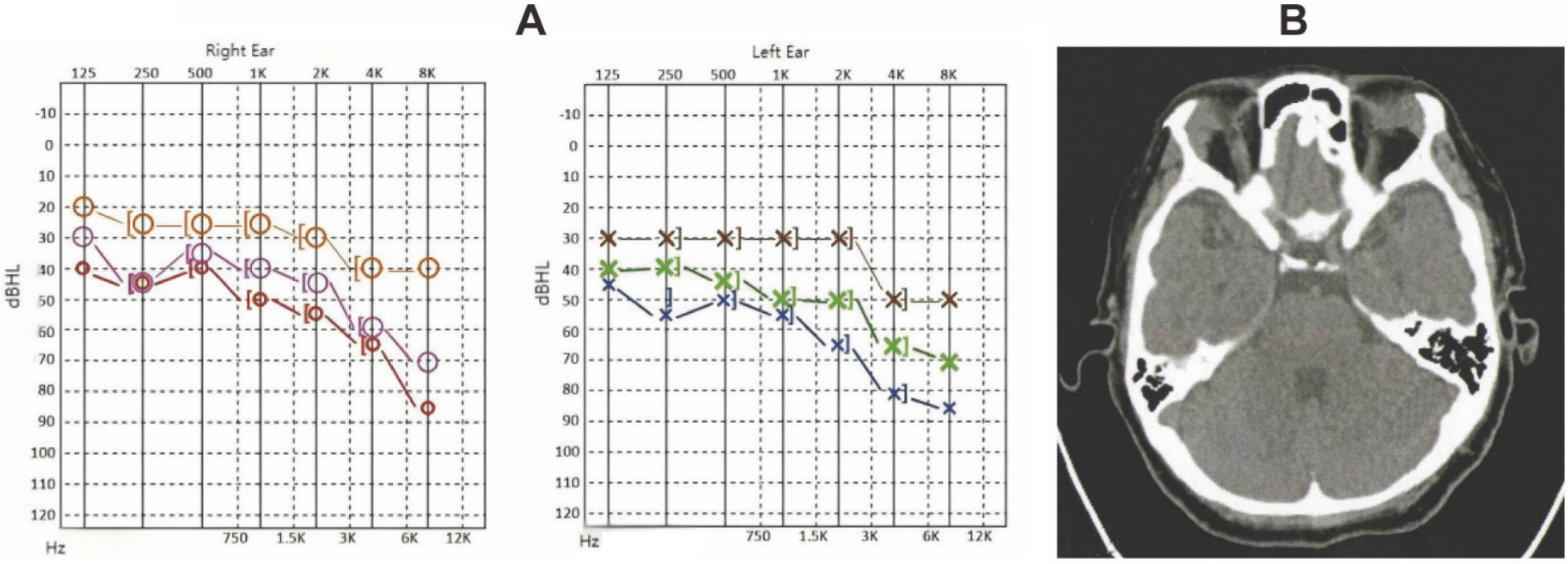
** The characterization of case-3.** A. Hearing curves before and after treatment for Case 3 (red and blue represent pre-treatment, pink and green represent 4 weeks post-treatment, orange and brown represent 16 weeks post-treatment). B. PET-CT findings for Case 3 show bilateral patent mastoid antrums with no soft tissue density shadows, intact ossicular chains, and normal structures of bilateral cochlea.

Laboratory findings included an ANA titer of 1:100, elevated serum IgG4 levels (32300 mg/L; normal 80-1400 mg/L), and negative rheumatoid factor and ANCA tests. Audiometry revealed symmetric bilateral sensorineural hearing loss with pronounced high-frequency impairment; right ear air and bone conduction thresholds were 48 dB, and left ear thresholds were 57 dB. PET-CT indicated enlargement and increased metabolic activity in bilateral submandibular, parotid, and lacrimal glands (Figure 3A-B), suggestive of IgG4-related systemic involvement.

The patient received intravenous methylprednisolone (40 mg daily) and tripterygium glycosides (20 mg three times daily), followed by a tapering regimen. After four weeks, submandibular gland size decreased, and serum IgG4 levels dropped significantly to 2800 mg/L. Sixteen-week follow-up audiometry showed substantial improvement, with air conduction thresholds at 27 dB for the right ear and 30 dB for the left, reflecting marked recovery in both low and high-frequency ranges.

## Discussion

This study provides a comprehensive analysis of three cases of autoimmune inner ear disease (AIED), focusing on the clinical, audiological, and immunological profiles of patients, as well as their responses to corticosteroid and immunosuppressive therapies. AIED, a condition characterized by immune-mediated inner ear inflammation leading to progressive sensorineural hearing loss (SNHL) 5, 26, poses diagnostic and therapeutic challenges due to its complex pathophysiology and varied clinical presentation 27. The findings from our cases, along with an extensive literature review, highlight several key aspects of AIED pathogenesis, diagnosis, and management strategies.

In each of the presented cases, SNHL was a primary clinical feature, often accompanied by tinnitus or vestibular symptoms 28. The pattern of hearing loss, primarily high-frequency sensorineural 29, aligns with previous studies indicating that AIED often leads to progressive, symmetric SNHL with a higher prevalence in high frequencies. Case 1, which involved a patient with rheumatoid arthritis, and Case 3, diagnosed with IgG4-related disease, both exhibited positive autoimmune markers, including antinuclear antibodies (ANA) and anti-cyclic citrullinated peptide antibodies (ACCP), consistent with reports of systemic autoimmune involvement in AIED 30. These cases underscore the frequent association of AIED with systemic autoimmune diseases, supporting the hypothesis that immune dysregulation plays a pivotal role in inner ear pathology.

The presence of specific and non-specific autoantibodies in AIED has been widely documented, suggesting that these antibodies may serve as biomarkers for diagnosis and disease activity monitoring. Studies have identified several candidate autoantigens, such as heat shock protein 70 (HSP70), collagen type II, and cochlin, which may trigger immune responses in susceptible individuals 31-33. HSP70, in particular, has been shown to have diagnostic value in AIED due to its elevated levels during immune-mediated cochlear injury 33. Matsuoka *et al.* reported HSP70 antibody sensitivity at 54.5% and specificity at 42.9% for AIED diagnosis 34, highlighting its potential role as a diagnostic marker. Our findings align with these observations, as patients in Cases 1 and 3 exhibited positive ANA and ACCP, underscoring the involvement of both specific and non-specific antibodies in AIED 35.

The pathogenesis of AIED remains incompletely understood, with multiple mechanisms proposed to explain immune-mediated cochlear damage. The “bystander effect” suggests that cytokine release, including IL-1 and TNF, following inner ear damage may incite secondary immune responses. Alternatively, molecular mimicry between inner ear antigens and pathogen-derived antigens may lead to cross-reactive immune responses, particularly in patients with preceding infections 36. Another hypothesis posits immune intolerance, where damage to the inner ear leads to the exposure of hidden antigens, subsequently triggering an autoimmune response. Genetic predisposition also likely plays a role in AIED, with certain gene expressions potentially activating autoimmunity in the inner ear 37, 38.

A critical challenge in AIED management is the timely initiation of effective therapy. Glucocorticoids are the first-line treatment, with approximately 70% of AIED patients showing sensitivity to corticosteroids 39-41. In our cases, all patients received corticosteroid therapy, with variable responses reflecting the heterogeneity of AIED. Case 1 experienced significant improvement in high-frequency hearing thresholds following a three-month corticosteroid regimen, while Case 2, with more severe baseline hearing loss, showed limited improvement in the left ear. This observation aligns with previous studies suggesting that early intervention and shorter disease duration are associated with better hearing recovery outcomes. However, the limited efficacy of corticosteroids in certain cases necessitates alternative therapeutic options.

Immunosuppressive agents, including methotrexate, azathioprine, and mycophenolate mofetil, have been employed as adjunctive therapies in AIED cases resistant to corticosteroids 42, 43. In our study, patients were treated with adjunctive immunosuppressive agents tailored to their systemic autoimmune conditions; for instance, Case 1 received leflunomide due to rheumatoid arthritis, while Case 2, diagnosed with relapsing polychondritis, was treated with azathioprine alongside corticosteroids. Immunosuppressive therapy helped stabilize symptoms during corticosteroid tapering, reducing the risk of recurrence and allowing for a more controlled reduction of steroid dosage. This supports the role of immunosuppressants as valuable corticosteroid-sparing agents in AIED, particularly in cases where corticosteroids alone fail to achieve sustained hearing improvement or where prolonged steroid use is contraindicated due to adverse effects.

Biologic therapies targeting specific immune pathways have emerged as promising treatment options in refractory AIED 44, 45. Agents such as infliximab (a TNF inhibitor), tocilizumab (an IL-6 receptor antagonist), and rituximab (a CD20 monoclonal antibody) have demonstrated efficacy in controlling systemic autoimmune conditions with otologic manifestations 46. Although biologics were not administered in our cases, existing literature indicates that biologics could play a role in AIED management, particularly in patients with concomitant autoimmune diseases that are unresponsive to conventional immunosuppression. Intratympanic injection of biologics represents another novel approach, allowing targeted delivery to the cochlea while minimizing systemic exposure. However, further studies are needed to establish standardized guidelines for biologic use in AIED and evaluate their long-term safety and efficacy.

The diagnostic criteria for AIED remain a subject of debate, as AIED often presents without distinct biomarkers or radiographic findings 47. According to the 1994 criteria established by the National Symposium on Autoimmune Inner Ear Disease, AIED diagnosis is based on rapidly progressive, fluctuating, bilateral or unilateral SNHL accompanied by vestibular symptoms, serological immune abnormalities, and responsiveness to immunosuppressive therapy 48. However, the heterogeneous presentation of AIED, including cases with unilateral hearing loss or lack of vestibular symptoms, suggests that the current diagnostic criteria may not capture the full spectrum of disease. Our cases illustrate the variability in clinical presentation, with Case 1 showing bilateral symmetric hearing loss and vestibular symptoms, while Case 2 exhibited asymmetric hearing loss with severe high-frequency loss in one ear. These findings highlight the need for revised diagnostic criteria incorporating broader clinical phenotypes and the possible role of serological biomarkers.

The role of specific autoantibodies in AIED has been increasingly recognized, though the full spectrum of pathogenic antibodies in AIED remains incompletely mapped 49, 50. In addition to ANA and ACCP, other autoantibodies, such as those against type II collagen, cochlin, and HSP70, have been implicated in AIED pathogenesis. Anti-HSP70 antibodies, in particular, are thought to contribute to immune-mediated cochlear injury through a mechanism involving molecular mimicry and cross-reactive immune responses. Recent studies have also shown elevated serum P0 protein antibodies in AIED patients 51, suggesting that anti-P0 antibodies could serve as additional markers of inner ear autoimmunity. The detection of these antibodies could improve AIED diagnosis and facilitate early therapeutic intervention. However, the limited availability of commercial assays for these antibodies restricts their routine clinical application, indicating a need for further research to develop accessible diagnostic tools.

A notable finding in our cases is the association between AIED and systemic autoimmune diseases. In Case 1, the patient's longstanding rheumatoid arthritis and high titers of ANA and ACCP suggest that systemic inflammation may contribute to inner ear involvement. Similarly, Case 3's IgG4-related disease highlights the potential link between systemic IgG4-mediated inflammation and cochlear pathology. IgG4-related disease has been associated with multi-organ involvement, and emerging evidence suggests that it may also affect the inner ear, leading to progressive hearing loss through chronic inflammation and fibrosis 52, 53. These cases underscore the importance of a multidisciplinary approach in AIED management, where rheumatologists and otolaryngologists collaborate to address systemic and otologic manifestations of autoimmune disease. This interdisciplinary approach is essential for optimizing patient outcomes, particularly in complex cases with multi-system involvement.

In summary, this study demonstrates that AIED is a multifaceted disorder with variable clinical manifestations, immunological profiles, and treatment responses. The findings underscore the importance of early diagnosis and a tailored therapeutic strategy, combining corticosteroids, immunosuppressive agents, and, when necessary, biologics. While corticosteroids remain the mainstay of therapy, the addition of immunosuppressants and potentially biologics offers hope for patients with refractory disease or those at risk of recurrence. Future research should focus on elucidating the immunopathogenic mechanisms underlying AIED, identifying novel biomarkers for more precise diagnosis, and developing standardized treatment protocols that incorporate recent advances in biologic therapy. The potential role of personalized medicine in AIED should also be explored, as it may allow for individualized treatment strategies that are tailored to each patient's unique immunologic and clinical profile. For example, patients with specific autoantibodies or with systemic autoimmune disease manifestations may benefit from early immunosuppressive or biologic therapy, while those with isolated inner ear involvement may respond adequately to corticosteroids alone.

Despite these advances, several challenges remain in the management of AIED. The identification of reliable biomarkers for AIED diagnosis is still limited, and the role of specific autoantibodies, such as anti-HSP70, anti-cochlin, and anti-collagen type II, requires further validation in larger patient cohorts. The development of accessible and standardized assays for these markers would significantly enhance the ability to diagnose AIED early and monitor disease progression. Additionally, studies are needed to determine the optimal duration and dosing of corticosteroids and immunosuppressants, as prolonged exposure to these agents can lead to significant adverse effects. Research into the efficacy and safety of biologics, particularly their long-term effects and potential for inner ear preservation, is essential to establish these therapies as viable options in routine AIED treatment.

Lastly, there is a pressing need for more robust clinical trials and cohort studies to refine the diagnostic criteria for AIED and to establish evidence-based treatment algorithms. Given the rarity of AIED, multicenter collaborations and registries could facilitate data collection and provide insights into the natural history, prognostic factors, and treatment outcomes in a larger, more diverse patient population. The heterogeneity of AIED presentations highlights the need for flexible diagnostic criteria that can accommodate varying patterns of hearing loss, vestibular symptoms, and systemic immune involvement.

In conclusion, AIED represents a complex autoimmune disorder with diverse clinical manifestations and treatment challenges. Our study supports the utility of a combined approach, integrating corticosteroids, immunosuppressive agents, and potentially biologics, for achieving better clinical outcomes. A deeper understanding of AIED pathogenesis, coupled with advances in diagnostic biomarker development, will be pivotal in shaping future strategies for the timely and effective management of this condition. Continued interdisciplinary collaboration, both in clinical practice and research, is essential for advancing the care of AIED patients and mitigating the impact of this debilitating disease on patients' quality of life.

## Funding

This study is supported by the Key Integrated Traditional Chinese and Western Medicine Specialty Construction Project in Tianjin.
